# Theoretical-Experimental Analysis to Elucidate the Mechanism of Action of Novel Anabolic Agents

**DOI:** 10.3390/molecules30224486

**Published:** 2025-11-20

**Authors:** Israel Quiroga, Maura Cardenas-Garcia, María Guadalupe Hernández-Linares, Gabriel Guerrero-Luna, Fermín Flores-Manuel

**Affiliations:** 1 Department of Life and Health Sciences, Universidad Popular Autónoma del Estado de Puebla, 13 Poniente No. 1927, Barrio de Santiago, Puebla 72410, Mexico; israel.quiroga@upaep.mx; 2 Laboratorio de Fisiología Celular, Facultad de Medicina, Benemérita Universidad Autónoma de Puebla, Puebla 72420, Mexico; 3 Laboratorio de Flujo Continuo y Fotoquímica, Centro de Química, Instituto de Ciencias, Benemérita Universidad Autónoma de Puebla, Puebla 72570, Mexico; gabriel.guerrerolu@correo.buap.mx; 4 Laboratorio de Investigación, Herbario y Jardín Botánico Universitario, Benemérita Universidad Autónoma de Puebla, Puebla 72570, Mexico

**Keywords:** anabolic signalling, hydrazone, cytometry, molecular docking

## Abstract

Research into muscle tissue pathologies offers great opportunities for new pharmaceutical agents. Current therapies, including corticosteroids and immunosuppressants, have limited efficacy and significant adverse effects. In this context, steroidal hydrazone compound **4d** was investigated for its ability to promote muscle growth and regeneration as a potential anabolic and regenerative modulator. Flow cytometry analysis showed that **4d** significantly increases cell populations in S phase, indicating a strong proliferative stimulus in pathways regulated by TNF-α, AKT, MAFbX, and SMAD2/3. Molecular docking studies showed that **4d** shares strong interactions with the known MasR agonist (EP-2825), exhibiting a higher predicted binding affinity. Furthermore, **4d** demonstrated the ability to interact with ACVR1/2A receptors, mimicking the binding profiles of known antagonists and potentially inhibiting myostatin/SMAD signaling. Taken together, experimental and computational evidence supports a dual-mechanistic model in which **4d** promotes muscle proliferation and regeneration by (**1**) activating the MasR–PI3K/AKT/mTOR axis and (**2**) inhibiting the ACVR1/2A–SMAD pathway, counteracting the action of myostatin. These findings position compound **4d** as a promising therapeutic candidate against muscle wasting disorders, including cancer-related cachexia, by inducing a robust and multifactorial anabolic response.

## 1. Introduction

Muscle tissue plays a vital role not only in locomotion but also in systemic metabolic regulation through its endocrine function [1]. Disorders affecting muscle integrity, especially those involving respiratory and cardiac musculature, pose significant clinical risk [2]. Therapeutic options remain limited, often relying on corticosteroids and immunosuppressants [3,4,5,6] with variable efficacy, high costs, and considerable side effects. These limitations underscore the need for novel pharmacological strategies grounded in a deeper understanding of the molecular and cellular mechanisms governing muscle tissue homeostasis and regeneration.

With this rationale, we identified a pressing need to develop and evaluate novel agents capable of counteracting muscle wasting and promoting regeneration in neuromuscular disorders such as spinal muscular atrophy and cachexia. In our previous work [7], we designed, synthesized, and evaluated a new series of spirostanic hydrazone derivatives with anabolic activity. Among these, compound **4d** showed the most promising biological profile, significantly increasing both skeletal muscle cells and their protein content. However, the molecular mechanism underlying this activity remained unclear. The present study, therefore, aimed to elucidate the potential molecular targets and signaling pathways modulated by compound **4d**, combining in silico docking analyses with functional cellular assays to explore its role in muscle anabolism and homeostasis.

**Figure 1:**
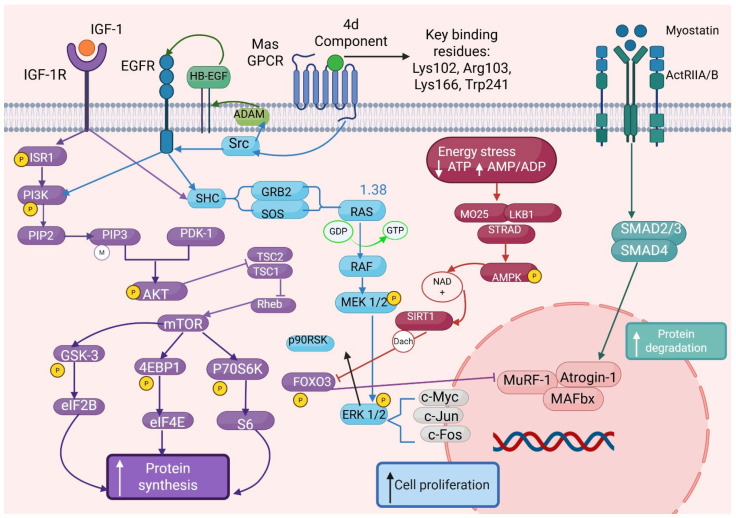
Signaling pathways involved in muscle metabolism and mechanistic integration of compound **4d**-mediated Mas receptor (MasR) signaling in the regulation of muscle anabolism and cellular proliferation. The diagram depicts the molecular pathway by which compound **4d** targets MasR through specific binding interactions with key residues Lys102, Arg103, Lys166, and Trp241, as identified by molecular docking studies. Upon ligand binding, MasR activation initiates G-protein-coupled signaling cascades through both Gq/11 and Gi/o pathways, subsequently modulating the PI3K/AKT/mTOR signaling axis. This activation leads to enhanced protein synthesis machinery engagement (S6K1, 4EBP1, eIF2B) and dramatic increases in S-phase cell populations, promoting cellular proliferation. The network illustrates the synergistic cross-talk between MasR signaling and traditional myokine pathways, including myostatin/ActRIIA/B-mediated regulation, demonstrating convergent anabolic effects. Additional regulatory nodes include FOXO transcription factors, autophagy modulation, and protein degradation pathways (ubiquitin-proteasome system). The integrated signaling network reveals novel therapeutic opportunities for muscle anabolism through MasR-targeted interventions. Phosphorylation events, protein–protein interactions, and inhibitory regulations are indicated by connecting lines and regulatory symbols, with the cellular membrane represented by the vertical dashed boundary.

Muscle growth and atrophy are tightly regulated by a balance between anabolic and catabolic signaling pathways. The PI3K/Akt/mTOR signaling pathway, typically activated by insulin-like growth factor 1 (IGF-1), plays a central role in promoting protein synthesis and hypertrophy through a cascade of phosphorylation events that activate both PI3K-Akt-mTOR and Ras-MAPK-ERK pathways [8,9,10,11]. Within this cascade, class I phosphoinositide 3-kinases (PI3Ks) generate the second messengers PIP2 and PIP3, critical for Akt membrane recruitment and activation. Akt exerts broad anabolic effects, including inhibition of skeletal muscle atrophy by activating mTOR and simultaneously inhibiting the transcriptional activity of key atrogenes—MuRF1 and MAFbx (Atrogin-1)—via phosphorylation and nuclear exclusion of FOXO transcription factors (Figure 1).

Emerging evidence indicates that the Mas receptor (MasR), G protein-coupled receptor activated by angiotensin-(1–6,8), may also engage this anabolic network, contributing to enhanced muscle regeneration, anti-atrophic effects, and cytoprotection through convergence on the PI3K-Akt-mTOR pathway [8]. Conversely, the myostatin/ActRIIB/SMAD pathway acts as a potent negative regulator of muscle mass, promoting atrophy through transcriptional activation of catabolic genes as part of a feedback mechanism aimed at limiting excessive muscle expansion and metabolic demand [8,9,10]. In addition to the canonical SMAD pathway, myostatin signaling also engages non-canonical pathways, including p38/MAPK, Erk1/2 MAPK via Ras, PI3K/Akt/GSK-3β, and JNK signaling cascades [11]. Inflammatory cytokines like TNF-α further exacerbate muscle wasting via two distinct cell surface receptors, TNFR1 and TNFR2, which differ in their intracellular signaling motifs. TNFR1 contains a death domain driving apoptotic and catabolic programs through NF-κB activation, while TNFR2 predominantly signals through non-canonical NF-κB and PI3K-dependent pathways to support cell survival [12,13,14,15,16].

## 2. Results

**Figure 2:**
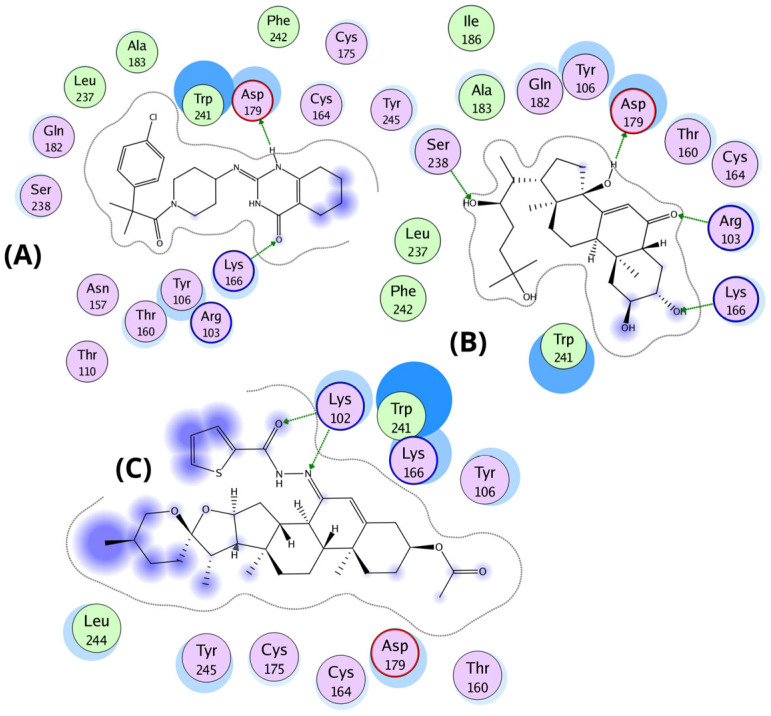
Chemical structure of the molecules used in the molecular docking analysis. (**A**) Structure of the co-former present in the co-crystal of the protein deposited in PDB (PDB ID: 9DQH). (**B**) Steroidal compound ecdysone with anabolic properties in preclinical models. (**C**) Chemical structure of compound **4d** with strong binding affinities in the ligand-binding domains of both IGFR and MasR.

In this study, we employed in silico molecular docking to evaluate the interaction of steroidal compounds with key receptors involved in muscle homeostasis: IGFR, MasR, ActRIIB, and TNFR1/2. Our findings reveal favorable binding affinities across anabolic and catabolic pathways, suggesting that these molecules may function as dual agonists or modulators capable of promoting muscle growth or counteracting atrophy, depending on the context of receptor engagement. These insights lay the foundation for further experimental validation and the development of targeted therapies for muscle dysfunction with improved efficacy and safety profiles. Natural steroidal compounds such as ecdysone (Figure 2B) and diosgenin have shown anabolic properties in preclinical models, including enhanced protein synthesis and protection against muscle atrophy [17,18]. Recent evidence suggests that part of their beneficial effects may be mediated through non-classical steroid pathways, including activation of the Mas receptor (MasR), a component of the renin–angiotensin system known to promote muscle regeneration and anti-inflammatory responses [8,9,10,11,12,13,14,15,16,17,18,19].

Based on structural similarities and docking-based binding affinity, in silico molecular docking studies performed in this work revealed that compound **4d** exhibits strong binding affinities at the ligand-binding domains of both IGFR and MasR, raising the possibility that this molecule may act as a dual agonist or modulator capable of triggering downstream anabolic signaling (Figure 2C). Its favorable interactions with MasR and IGFR suggest that **4d** may modulate key signaling pathways implicated in muscle protein synthesis and hypertrophy [8,9,20,21,22,23,24,25]. We hypothesize that compound **4d** could act as a MasR agonist, leading to activation of the PI3K/Akt/mTOR signaling pathway, thereby counteracting muscle wasting in catabolic conditions such as cachexia. Additionally, computational molecular docking analyses revealed that compound **4d** exhibits high binding affinities not only toward IGFR and MasR but also at the ligand-binding site of ActRIIB, suggesting a potential capacity to modulate or antagonize myostatin signaling. This potential functional modulation may position **4d** as a pharmacological candidate for muscle-preserving interventions as a dual anabolic and anti-catabolic agent.

### 2.1. Flow Cytometry Analysis

**Figure 3:**
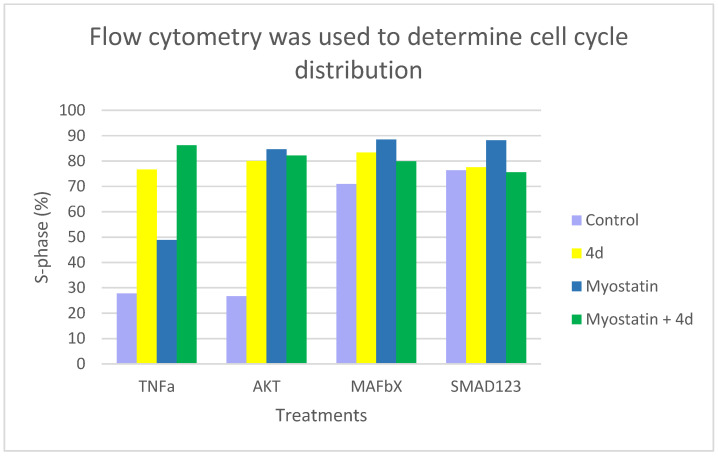
Cell cycle distribution was assessed by flow cytometry after staining with propidium iodide. The percentage of S-phase cells was quantified in subpopulations positive for TNFα, AKT, MAFbX, and SMAD1/2/3. These measurements were taken after treatment with control medium (light purple), compound **4d** (yellow), myostatin (blue), or a combination of myostatin and **4d** (green). Treatment with compound **4d** markedly increased the proportion of S-phase cells in TNFα- and AKT-positive populations. When myostatin and **4d** were combined, additive effects were observed in TNFα-positive cells, but attenuated responses were noted in SMAD-positive subpopulations. Bars represent mean values from two independent experiments.

Flow cytometric analysis revealed that treatment with compound **4d** significantly modulated cell cycle distribution across all experimental conditions, indicating potent anabolic effects. The most consistent and prominent observation was a substantial increase in the S-phase population, which reflects elevated DNA replication and cellular anabolic activity. In the TNF-α signaling pathway, **4d** induced a 177% increase in the proportion of S-phase cells—from 27.73% in untreated controls to 76.67%—accompanied by a notable reduction in the G1-phase population (from 46.59% to 13.87%) (Graphics S3, S8, S9 and S16). This shift suggests a strong proliferative and anabolic stimulus capable of counteracting catabolic signaling. The AKT pathway exhibited the most profound response to **4d**. The cell cycle profile changed from a typical distribution (52.46% G1, 26.55% S) to a hyper-anabolic profile, with 80.05% of cells in S-phase and only 2.18% in G1. This represents a 201% increase in S-phase occupancy, indicating that AKT signaling may be a primary target of **4d**-mediated activation (Graphics S1, S2, S10 and S11). In the MAFbX signaling pathway, where baseline anabolic activity was already elevated (70.94% S-phase), treatment with **4d** further increased the S-phase proportion to 83.45%, reinforcing the anabolic potential of the compound even under conditions of high baseline synthetic activity (Graphics S4, S5, S12 and S13). The SMAD2/3 pathway also displayed a high basal S-phase percentage (76.39%), which remained consistently elevated after 2 and 4 days of treatment (77.90% and 77.53%, respectively), suggesting a stabilizing rather than amplifying role of **4d** in SMAD-mediated signaling. Importantly, co-treatment with myostatin across all pathways maintained or enhanced S-phase induction (Graphics S6, S7, S14 and S15). The most significant synergistic effects were observed in the SMAD2/3 (88.21%) and MAFbX (88.48%) signaling pathways. In the TNF-α model, the combination of **4d** and myostatin resulted in a maximal increase in S-G2/M phase populations (86.20%), highlighting a robust cooperative anabolic effect despite the presence of a catabolic signal. Overall, quantitative analysis of G1, S, and G2/M phase distributions (detailed in Table S1) confirmed the reproducible anabolic effects of **4d**. Computational models of cell cycle transitions under anabolic stimulation corroborated the observed profiles. Among all tested conditions, the AKT and TNF-α pathways emerged as the most responsive, supporting their relevance in the anabolic mechanism underlying **4d** action (Figure 3).

### 2.2. Molecular Docking

#### 2.2.1. Molecular Docking with MasR in Agonist-Bound Conformation

Molecular docking studies revealed that ecdysone, and compound **4d** share key binding interactions with the known MasR agonist EP-2825 within the active site of the human Mas receptor (PDB ID: 9DQH) [26]. The interaction profiles suggest a conserved mechanism of binding, with emphasis on electrostatic and hydrogen-bond interactions involving critical residues. All three ligands engage in electrostatic interactions with residues K102, R103, K166, and D179, which appear essential for agonist activity. Ecdysone maintains canonical electrostatic interactions via its carbonyl group with K102 and R103, and additionally forms hydrogen bonds with D179 through its hydroxyl group. Similarly, compound **4d** retains the carbonyl-mediated interactions with K102 and R103 but lacks direct hydrogen bonding with D179. Instead, a close interaction between K166 and D179 allows K166 to form a compensatory hydrogen bond with the spiroketal group of **4d**.

In the central region of the ligands, both ecdysone and **4d** penetrate more deeply into the binding pocket, interacting with Y106, N157, and W241. Notably, ecdysone and EP-2825 exhibit hydrophobic interactions with A183 and I186, whereas **4d** is positioned approximately 5 Å away from these residues. Distal interactions include a hydrogen bond between ecdysone and S238, while **4d** interacts via hydrogen bonding with N157. Previous mutagenesis studies indicate that alanine substitutions of N157 and Q182 slightly enhanced MasR activation by EP-2825 [26]. However, these substitutions may not benefit ligands like **4d** or ecdysone that rely on specific polar contacts at these positions. Extending toward the receptor’s deeper pocket, ecdysone forms direct contact with F242, and one of its hydroxyl groups is located within 4 Å of Y245, suggesting potential p-stacking or hydrogen bonding interaction.

**Figure 4:**
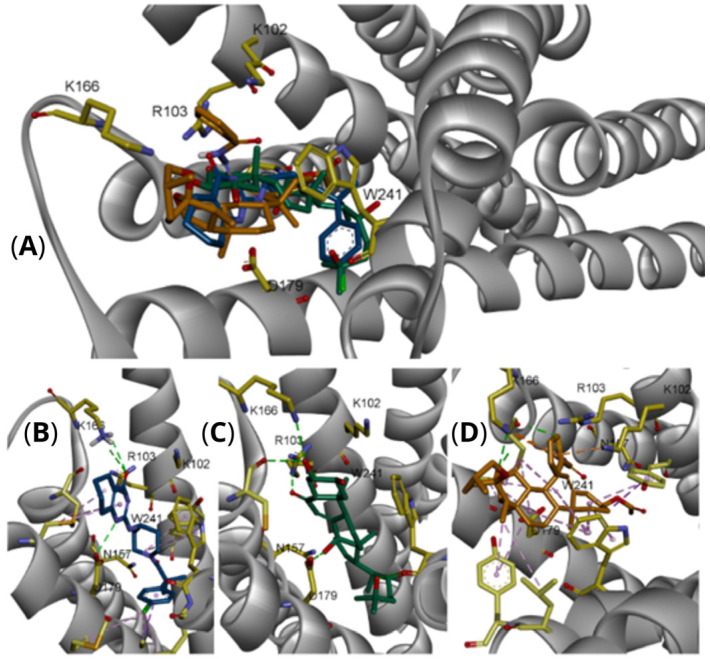
Binding interactions of EP-2825, ecdysone, and compound **4d** within the agonist-binding pocket of the Mas receptor (PDB: 9DQH). (**A**) Superposition of EP-2825 (yellow carbons), ecdysone (cyan carbons), and **4d** (green carbons) within the MasR binding pocket. Key residues (gold sticks) are labeled, and hydrogen bonds are represented as dashed green lines. (**B**) Binding pose of the co-crystallized agonist EP-2825, highlighting canonical interactions with key residues (Lys102, Arg103, Lys166, Asn157, Asp179, and Trp241). (**C**) Binding mode of ecdysone, preserving charge interactions with Lys102 and Arg103 and forming additional hydrogen bonds with Asp179. (**D**) Binding mode of compound **4d**, showing alternative hydrogen bonding involving Lys166 and the hemiacetal group, alongside hydrophobic contacts with Trp241 and Asn157.

In contrast, **4d** forms hydrophobic interactions with Y245, potentially contributing to its partial agonist profile. Finally, binding free energy estimations support the observed structural interactions: compound **4d** (−11.5 kcal/mol) exhibited the strongest affinity, followed by EP-2825 (−10.5 kcal/mol) and ecdysone (−9.0 kcal/mol). These results suggest that compound **4d**, although structurally distinct, shows the strongest predicted binding affinity—potentially attributed to its larger molecular framework—highlighting its relevance for further investigation as a MasR modulator. Additionally, docking of the known antagonist ligand from the 8DWH structure into the agonist-bound conformation of MasR (9DQH) revealed suboptimal binding. Its optimal binding pose exhibited a binding free energy of −8.0 kcal/mol, driven primarily by electrostatic interactions between its sulfide group and the positively charged residues Lys102 and Arg103, though this configuration appeared in only 11 out of 250 poses. In contrast, the most populated cluster (185/250 poses) displayed a lower binding affinity (−7.0 kcal/mol) and was characterized by interactions between Lys166 and the ligand’s hydrophobic bicyclic core, which engaged in an arene–arene interaction with Trp241—suggesting a distinct, likely inactive binding mode incompatible with agonist activity (Figure 4).

#### 2.2.2. Molecular Docking with MasR in Antagonist-Bound Conformation

Molecular docking simulations of diosgenin, compound **4d**, and ecdysone against the antagonist-bound conformation of MasR (PDB ID: 8DWH) revealed distinct binding behaviors compared to the reference antagonist compound 16. Notably, the key electrostatic interactions observed between compound 16 and residues Tyr99, Asp177, and particularly Glu157 were not recapitulated by either **4d** or ecdysone. Ecdysone retained similar binding features as observed in the agonist-bound conformation (9DQH), with the carbonyl group engaging Lys102 and Arg103, and its hydroxyl group forming hydrogen bonds with Asp179. This suggests a limited conformational adaptability of ecdysone across MasR states, possibly reflecting a preference for agonist-like binding.

In contrast, compound **4d** adopted a markedly different binding pose relative to its configuration in the agonist model. In this conformation, **4d** did not interact with the key antagonist-associated residues Tyr99 or Glu157, suggesting a reduced compatibility with the inactive receptor state. Binding energy estimations indicated comparable affinities: ecdysone exhibited a binding energy of −9.0 kcal/mol, albeit distributed across 16 clusters within a 2 Å RMSD threshold, reflecting a broader range of conformational poses. Compound **4d** followed closely with −8.0 kcal/mol and showed an increase in the number of clusters from 8 (in agonist conformation) to 16 in the antagonist model, indicating reduced convergence. Interestingly, compound 16 itself showed a relatively lower binding energy (−7.0 kcal/mol), despite being co-crystallized in this conformation, suggesting that its binding is stabilized more by specific interactions than overall binding affinity.

#### 2.2.3. Molecular Docking Results with ACVR1 and ACVR2A (Antagonist Conformation)

Consistent with known inhibitors such as dorsomorphin and LDN-193189, compound **4d** exhibited favorable binding affinities with both ACVR2A and ACVR1, yielding docking scores of −10.0 kcal/mol (PDB: 3Q4T) and −11.0 kcal/mol (PDB: 3H9R), respectively—comparable to those of dorsomorphin under identical conditions. In both receptors, compound **4d** forms key electrostatic interactions with lysine residues critical for ligand binding: Lys219 in ACVR2A and Lys235 in ACVR1. These residues are known to engage in salt bridges or hydrogen bonding with polar or charged groups of kinase inhibitors, thereby anchoring them within the ATP-binding pocket. Hydrophobic interactions were also prominent across both complexes. In ACVR2A, residues Ala199, Val206, Phe269, Leu329, and Ala339 interact with the planar, steroidal scaffold of **4d**, stabilizing its orientation within the binding site. Notably, Phe269, a residue frequently implicated in ligand selectivity, forms favorable contacts with the hydrophobic face of the ligand. A structure-based alignment using STAMP revealed that the topological equivalent of Phe269 in ACVR1 is Tyr285. In the ACVR1–**4d** complex, Tyr285 is positioned adjacent to the hemiacetal moiety of **4d**, potentially enabling either a hydrophobic interaction (via its aromatic ring) or a hydrogen bond through its hydroxyl group. Additional hydrophobic contacts are maintained with Leu343, Ala353, and Val222, which are spatial homologs to Leu329, Ala339, and Val206, respectively, among the surrounding residues in ACVR1.

Taken together, these results suggest that compound **4d** can favorably engage with the ligand-binding pockets of both type I and type II activin receptors through a combination of conserved electrostatic and hydrophobic interactions, closely mimicking the binding profiles of known antagonists. This dual-target affinity may underlie its ability to modulate myostatin/GDF8 signaling, as inferred from functional assays and downstream SMAD modulation. The binding mode of compound **4d**, which closely mimics key interactions of the BMP receptor inhibitor LDN-193189 with the Activin receptor type-2A (PDB: 3Q4T)—including hydrogen bonding with His270 and salt bridge interactions involving Lys219 and Glu232—suggests that **4d** may similarly inhibit myostatin (GDF8) signaling. This inhibition could underlie its capacity to counteract SMAD-mediated pathways and potentially promote functional differentiation of myoblasts, as reported for LDN-193189 in C2C12 cells.

#### 2.2.4. Molecular Docking to Less Probable Targets: TNFa and IGF-1 Receptors

To date, no crystal structures of IGF-1R bound to its endogenous agonists (IGF-1 or IGF-2) have been resolved at atomic resolution in functional conformations, and available models are limited to ectodomain cryo-EM fragments (e.g., PDB: 6PYH, 6JK8) [27,28]. Similarly, although crystal structures of TNF-a bound to TNFR2 exist (e.g., PDB: 3ALQ) [29], these involve the extracellular ligand-binding domains and do not represent agonist-bound functional conformations comparable to the MasR–EP-2825 complexes [26]. Therefore, the Mas receptor remains unique among these receptors in possessing high-resolution crystal structures co-crystallized with synthetic agonists.

To explore the selectivity profile of compound **4d**, additional docking studies were conducted with receptors traditionally involved in muscle wasting and anabolic signaling, namely tumor necrosis factor a (TNFa) and the insulin-like growth factor 1 receptor (IGF-1R). For TNFa, molecular docking was performed using the co-crystallized structure with an inhibitory ligand (PDB: 2AZ5). Compound **4d** was docked into the same binding site as the co-crystallized small-molecule inhibitor. However, **4d** failed to replicate the key charge and hydrogen bond interactions established by the reference compound, indicating a likely lack of competitive or allosteric binding capability at this site.

Similarly, the docking of **4d** to IGF-1R was evaluated using the available cryo-EM structure (PDB: 6PYH), which represents the ectodomain complex with its natural peptide ligand. Re-docking of IGF-1 yielded an expected highly favorable binding free energy (~−25 kcal/mol), reflective of its large interface and multiple specific contacts. In contrast, compound **4d** exhibited a modest binding affinity of –10 kcal/mol in the same site, insufficient to suggest a functional mimicry of the natural ligand’s interaction network. Moreover, the peptidic nature and size of the native ligands of both TNFa and IGF-1R, which establish extensive protein–protein interfaces, make it structurally implausible for a small molecule such as **4d** to engage these receptors in an agonistic manner analogous to their endogenous ligands. Taken together, these results support the conclusion that TNFa and IGF-1R are unlikely to be primary pharmacological targets of compound **4d**. While computational affinity values are non-negligible, the absence of key anchoring interactions and the lack of supportive structural mimicry argue against a biologically relevant role for **4d** at these receptors.

## 3. Discussion

The present study integrates in vitro and in silico evidence to propose that compound **4d** exerts anabolic effects on skeletal muscle cells through dual engagement of the Mas receptor (MasR) and inhibition of activin receptors ACVR2A and ACVR1. Flow cytometry revealed that **4d** significantly increased the proportion of S-phase cells across several signaling contexts, with the most pronounced proliferative effects observed in AKT and TNFα pathways, suggesting a strong pro-proliferative and potentially pro-hypertrophic effect.

### 3.1. MasR-Mediated Proliferative Signaling

The proliferative response to **4d** appears to be partially mediated through MasR activation, a G protein-coupled receptor involved in muscle regeneration and cytoprotection via the PI3K/AKT/mTOR pathway. The significant increase in the proportion of S-phase cells expressing proliferation-associated proteins (AKT and TNFα) following **4d** treatment, compared to controls, suggests that **4d** may exert its effects through these signaling pathways. Supporting this hypothesis, previous studies indicate that ecdysone—a steroid prohormone of 20-hydroxyecdysone with structural similarity to **4d**—induces anabolic effects by activating the Mas receptor (MasR) [30,31,32,33,34]. Molecular docking studies showed that **4d** may adopt stable binding poses at the MasR agonist site (PDB: 9DQH), forming interactions similar to those of known agonists such as EP-2825 (experimental observations) [35] and ecdysone (modelled in this study) (Figure 4). Key interactions include electrostatic contacts with Lys102, Arg103, and Lys166, alongside hydrophobic interactions with Trp241 and Asn157. Notably, **4d** exhibits more favorable binding energies than the reference agonist, suggesting two key implications: (1) **4d** may function as a MasR agonist, potentially activating anabolic signaling via the AKT pathway, and (2) despite its distinct interaction profile, **4d** displays the highest predicted binding affinity among tested ligands; this enhanced affinity likely stems from its extended molecular structure, which enables additional interaction points. Based on both functional (AKT activation by MasR) and structural (possible affinity of small ligands for the receptor) evidence, it is plausible that **4d** promotes proliferation consistent with the robust AKT-associated proliferative effects observed experimentally, with a potential mechanistic link between **4d** and proliferative signaling, validating the hypothesis that **4d** functions as a MasR agonist [36,37,38]. Further supporting its potential as a MasR modulator worthy of further investigation.

### 3.2. Antagonism of Myostatin/SMAD Signaling

Treatment with **4d** did not modify the proportion of S-phase cells expressing SMAD1/2/3, indicating that this pathway is not directly involved in the proliferative response induced by **4d**. In AKT-, MAFbX-, and SMAD-positive subpopulations, myostatin alone elicited stronger proliferative responses than **4d**. Co-treatment with myostatin and **4d** attenuated these effects, and in SMAD-positive cells the response closely resembled that of the control (Figure 3). These findings suggest that **4d** may interfere with myostatin signaling in a context-dependent manner, likely through both direct and indirect inhibition of the SMAD pathway. This observation is consistent with in silico docking results. Docking analyses revealed that **4d** mimics critical interactions of established BMP inhibitors such as LDN-193189 within both ACVR2A (PDB: 3Q4T) and ACVR1 (PDB: 3H9R) binding sites [39]. Specifically, **4d** preserves critical contacts such as hydrogen bonding with His270 and salt bridge mimicry involving Lys219 and Glu232; in addition, hydrophobic interactions with conserved residues includes Phe269/Tyr285 and Leu329/Leu343. This points to a possible antagonistic or modulatory role of **4d** on myostatin signaling. Taken together, these findings support the hypothesis that **4d** acts as a dual inhibitor of ACVR1/2A, thereby attenuating SMAD-mediated signaling in a context-dependent manner.

### 3.3. Context-Dependent Myostatin Interactions

Co-treatment experiments with myostatin revealed context-dependent outcomes. While myostatin is classically characterized by anti-anabolic effects [40], in neonatal primary muscle cells it elicited differential responses depending on the signaling context, producing additive effects in the case of TNF-α, but proliferative responses in AKT-, MAFbX-, and SMAD-positive cells (stronger than those elicited by **4d**). In SMAD1/2/3-expressing cells, myostatin alone increased S-phase activity; however, co-treatment with **4d** produced a partial attenuation of this effect. This phenomenon aligns with previous reports indicating that myostatin may exhibit context-dependent [10,41] functions in neonatal muscle cells, potentially influenced by immature SMAD signaling, epigenetic permissiveness for proliferation, or crosstalk with AKT and ERK pathways [10,22]. However, **4d** appears to disrupt the canonical myostatin/SMAD signaling axis. When administered in combination with myostatin, **4d** appears to attenuate the antiproliferative effect associated with SMAD activation by myostatin, suggesting possible functional interference, also observed in [9]. It is highly plausible that myostatin plays a biphasic and contextual role, acting as a proliferative modulator rather than an absolute inhibitor at this early stage [42,43,44]. The combination of **4d** and myostatin yielded additive effects in TNFα-positive cells but failed to exceed the individual effect of myostatin in AKT-positive populations, indicating that **4d** modulates pathway crosstalk in a context-dependent manner.

### 3.4. Perspectives and Limitations

Docking studies with the IGF-1 receptor (PDB: 6PYH) and TNFα (PDB: 2AZ5) revealed limited structural compatibility with natural ligands. IGF-1 docking exhibited substantially higher binding energy (−25 kcal/mol) compared to **4d** (−10 kcal/mol), while **4d** failed to recapitulate essential interactions of co-crystallized inhibitors within TNFα structures. These findings indicate that these receptors are unlikely to serve as primary mediators of the observed effects. Some hypotheses that cannot be ruled out with these results are that steroid compounds involved in anabolic effects such as sapogenins, ecdysone, or **4d** act as modulators of multiple targets. Additionally, the possibility that **4d** modulates supplementary signaling pathways; including a negative feedback loop of the ERK1/2 pathway, allowing a finer control of muscle cell proliferation in neonatal cells or a possible simultaneous inhibition of p38α MAPK [7], which in this neonatal cell type is related to the activation of differentiation programs via MyoD and MEF2, may contribute to maintaining cells in an immature proliferative state [45]. Although molecular dynamics simulations were not performed in the present work, the high convergence and stability of the docking clusters strongly support the reliability of the predicted binding mode. In future studies, we will conduct MD simulations to evaluate the dynamic stability and energetics of these interactions and perform targeted experimental validation, such as binding assays, to confirm the computational predictions— including receptor binding assays, gene expression profiling, and muscle regeneration models. Nonetheless, the combination of consistent functional effects and mechanistically plausible molecular interactions provides a strong foundation for further investigation of compound **4d** as a dual-acting modulator of muscle anabolic signaling and an activator of the AKT/mTOR pathway via MasR. Under a working model where **4d** activates MasR to engage Gi/o and Gq signaling—thereby recruiting PI3K and activating AKT/mTOR—and where myostatin signaling is contextually permissive in neonatal cells, the observed increases in S-phase in AKT- and TNFα-gated populations are parsimoniously explained. This model predicts that pharmacological blockade of MasR or PI3K should abrogate the proliferative effect of **4d**, whereas direct inhibition of ACVR1/2 should mimic **4d**’s attenuation of myostatin-induced SMAD phosphorylation.

## 4. Materials and Methods

### 4.1. Docking Preparation

The three-dimensional structures of all ligands were constructed from their respective SMILES representations using VEGA ZZ 3.2.4.29 [46], which generated initial Cartesian coordinates suitable for further refinement. Geometry optimization and charge calculation was subsequently carried out using the semiempirical PM7 method implemented in MOPAC 2016 [47], with input preparation and analysis performed through the GABEDIT 2.5.2 [48] graphical user interface. To simulate physiological conditions, implicit solvation was applied using the Generalized Born model [49], with water as the solvent (dielectric constant ε = 78.39) and a cavity formation radius set at 1.3 Å. All optimizations were conducted under a singlet electronic configuration, employing detailed bond analysis and stringent convergence criteria. This procedure ensured the generation of low-energy ligand conformations within a solvent-mimicking environment, appropriate for downstream molecular docking analyses. For back-docking validations involving co-crystallized ligands, no additional geometry optimization was performed. Instead, only partial atomic charges were calculated using the Gasteiger method to preserve the experimentally determined binding conformations and ensure compatibility with the docking algorithm.

The crystallographic structures of the target proteins were retrieved from the Protein Data Bank (PDB) [50] and prepared for molecular docking studies. Each structure was selected based on resolution, ligand-bound state, and relevance to the proposed signaling pathways. Tumor necrosis factor α (TNF-α)—PDB ID 2AZ5 [27] was selected as it contains TNF-α in complex with a small-molecule inhibitor, enabling identification of the canonical ligand-binding site for docking validation. It was evaluated as a control to rule out relevant affinity, not as evidence of a primary mechanism. Insulin-like growth factor 1 receptor (IGF1R)—PDB ID 6PYH [28] was used as the primary docking template, as it contains the receptor bound to its endogenous ligand IGF-1. To confirm the receptor’s active conformation and ligand-binding site plasticity, structural comparisons were performed with PDB IDs 6JK8 [51] and 5U8Q [52], both of which represent active or ligand-bound states. Activin receptor type IIA (ACVR2A)—PDB ID 3Q4T [39] was selected for docking because it provides high-resolution structural data in complex with the inhibitor LDN-193189. Comparative analyses with PDB IDs 2QLU [53] and 3H9R [54] were performed to assess conformational variability and ligand interaction patterns. Activin receptor type I (ACVR1)—PDB ID 3H9R [54] was chosen as the primary docking structure due to its co-crystallization with dorsomorphin, an inhibitor of the BMP pathway. Cross-comparison with PDB IDs 6EIX [25], 2QLU [53], and 3Q4T [39] was performed to evaluate conformational shifts and conserved binding determinants across the TGF-β superfamily receptors. Mas receptor (MasR)—The agonist-bound structure PDB ID [35] (co-crystallized with EP-2825) was used to validate docking procedures and identify key agonist-binding interactions. The antagonist-bound structure PDB ID 8DWH [55] (compound-16) was used for cross-validation and comparative analysis of antagonist-specific interactions. At the time of this study, no crystallographic structure of MasR in complex with the antagonist peptide A779 was available, precluding direct computational modeling of this specific receptor–ligand interaction. Prior to docking, all protein structures were prepared by removing water molecules, co-crystallized ligands, and non-essential ions, except where retention was necessary to preserve critical structural or catalytic features. Polar hydrogens were added and Gasteiger [56] charges were assigned to ensure compatibility with docking software using the MGL-Tools 1.5.6 software [57].

### 4.2. Molecular Docking Procedure

Molecular docking simulations were conducted using AutoDock 4.2.6 [57], employing the Lamarckian Genetic Algorithm (LGA) for conformational sampling and optimization. For each protein–ligand pair, grid maps were generated with a spacing of 0.375 Å and grid dimensions adapted to the binding site defined by the coordinates of the co-crystallized ligands. In the case of MasR, the grid box was intentionally enlarged to encompass both the agonist (PDB: 9DQH, EP-2825) [35] and antagonist (PDB: 8DWH, compound-16) binding regions, ensuring that both conformational states could be explored under identical spatial constraints to avoid bias. Cross-validation was performed by re-docking the co-crystallized ligands of each protein into their respective binding sites, confirming that the docking protocol reproduced experimentally observed binding poses within <2.0 Å RMSD. For MasR, docking results for **4d** were directly compared against reference agonist (EP-2825) [35] and antagonist (compound-16) binding profiles to validate positional and interaction consistency with known ligand–residue contacts. Predicted binding poses of **4d** and reference ligands were analyzed in PyMOL 2.6 and LigPlot+ 2.3 to identify hydrogen bonds, salt bridges, and hydrophobic contacts. Key interactions were then compared with those reported in the literature for each receptor–ligand complex, with particular emphasis on functionally critical residues such as Lys102, Arg103, Lys166, and Trp241 in MasR; Lys219 and Glu232 in ACVR2A; and Tyr285 in ACVR1. Literature concordance in residue engagement was used as an internal validation criterion to ensure biological plausibility of the docking results.

### 4.3. Flow Cytometry Analysis

Cell cycle distribution and protein expression were assessed by flow cytometry following propidium iodide (PI) staining. Cells were processed according to standard fixation and permeabilization protocols, and nuclear DNA content was quantified to determine the proportion of cells in G1, S, and G2/M phases. For pathway-specific analysis, the expression of pTNF-α (Santa Cruz Biotechnology, sc-133192, Santa Cruz, CA, USA), pSMAD1/2/3 (sc-7960), pMAFbX (sc-166806), and pAKT (sc-514032) was evaluated using non–phospho-specific antibodies to quantify total protein levels in cells corresponding to the four signaling contexts of interest. Flow cytometric acquisition was performed on a BD FACSCanto II system (BD Biosciences, San Jose, CA, USA), using forward scatter area (FSC-A) gating to exclude debris and doublets. A minimum of 10,000 events per sample were collected to ensure statistical robustness. Data are presented as the percentage of cells in each cell cycle phase calculated from the DNA content histograms. Results are expressed as the mean ± standard error (S.E.) from two independent, representative experiments. Cell cycle phase distributions are expressed as percentages of total viable cells. Fold-change calculations were performed relative to pathway-specific control conditions.

## 5. Conclusions

Collectively, these results support a dual-mechanism model wherein compound **4d** promotes skeletal muscle proliferation and potentially enhances hypertrophy through two complementary pathways: (1) activation of the MasR–AKT–mTOR axis, and (2) inhibition of the ACVR1/2A–SMAD pathway, effectively alleviating myostatin-mediated growth repression. These complementary actions establish **4d** as a promising therapeutic candidate for treating muscle-wasting disorders, including cancer-associated cachexia.

Mechanistic evidence demonstrates that the proliferative effect observed with compound **4d** exerts proliferative effects even in the absence of myostatin, as evidenced by an increased proportion of S-phase cells co-expressing AKT and SMAD1/2/3. This finding suggests that **4d** modulates key intracellular pathways through activation of anabolic signaling via the PI3K/AKT axis while concurrently attenuating SMAD-mediated pathways, which are typically downstream of myostatin. Although these observations are correlative, they support a mechanistic model in which **4d** interferes with myostatin signaling and promotes muscle cell proliferation.

Supporting molecular evidence from docking analyses further substantiates this hypothesis, revealing that **4d** binds to the Mas receptor at the same site as the known agonist EP-2825, forming critical interactions and exhibiting predicted binding affinities in the nanomolar range. These structural findings align with experimental data showing enhanced S-phase entry and AKT expression upon **4d** treatment, consistent with partial agonism at MasR and downstream activation of the PI3K/AKT/mTOR pathway.

Furthermore, **4d** appears to counteract the antiproliferative effects of myostatin, likely through inhibition of SMAD-dependent signaling. Collectively, **4d** elicits a robust anabolic response characterized by enhanced DNA synthesis and cell cycle progression. Its dual role in stimulating AKT/mTOR signaling and modulating myostatin-SMAD pathways highlights its potential as a candidate for combination therapy, potentially engaging novel feedback loops and receptor crosstalk mechanisms relevant to muscle regeneration and anti-cachexia strategies.

## 6. Patents

The steroidal hydrazone derivatives **4a** and **4d** are protected by patent applications MX/a/2025/012402 and MX/a/2025/012412, resulting from the work reported in this manuscript and in [7].

## Data Availability

The original contributions presented in this study are included in the article/Supplementary Material. Further inquiries can be directed to the corresponding author(s).

## Supplementary Materials

The following supporting information can be downloaded at: https://www.mdpi.com/article/10.3390/molecules30224486/s1, Table S1: Quantitative distribution of cell cycle-treated and signaled. Image S1: Quantitative distribution of cell cycle-treated and signaled. Graphic S1: Control-AKT; Graphic S2: Myostatin-AKT; Graphic S3: Myostatin-4d-TNFα; Graphic S4: Myostatin-MAFBX; Graphic S5: Control-MAFBX; Graphic S6: Control-SMAD123; Graphic S7: Myostatin-SMAD123; Graphic S8: Control-TNFα; Graphic S9: Myostatin-TNFα; Graphic S10: 4d-AKT; Graphic S11: Myostatin-4d-AKT; Graphic S12: 4d-MAFBX; Graphic S13: Myostatin-4d-MAFBX; Graphic S14: 4d-SMAD123; Graphic S15: Myostatin-4d-SMAD123; Graphic S16: 4d-TNFα.
