# Supplementary material for: Theoretical-Experimental Analysis to Elucidate the Mechanism of Action of Novel Anabolic Agents
Source: Molecules. 2025 Nov 20;30(22):4486. doi: 10.3390/molecules30224486 (PMC12654979; doi:10.3390/molecules30224486)
Supplement: Supplementary file 1 [file molecules-30-04486-s001.zip › molecules-3964345-supplementary.pdf]

Supplementary Materials

Theoretical-Experimental Analysis to Elucidate the Mechanism of Action of Novel Anabolic Agents

Israel Quiroga [1], Maura Cardenas-Garcia [2,*], María Guadalupe Hernández-Linares [3,4,*], Gabriel Guerrero-Luna [3,4] and Fermín Flores-Manuel [3,*]

[1] Department of Life and Health Sciences, Universidad Popular Autónoma del Estado de Puebla, Puebla 72410, Mexico; israel.quiroga@upaep.mx

[2] Laboratorio de Fisiología Celular, Facultad de Medicina, Benemérita Universidad Autónoma de Puebla, Puebla 72420, Mexico

[3] Laboratorio de Flujo Continuo y Fotoquí mica. Centro de Química, Instituto de Ciencias. Benemérita Universidad Autónoma de Puebla, Puebla 72570, Mexico

[4] Laboratorio de Investigación, Herbario y Jardín Botánico Universitario, Benemérita Universidad Autónoma de Puebla, Puebla 72570, Mexico; gabriel.guerrerolu@correo.buap.mx

* Correspondence: maura.cardenas@correo.buap.mx (M.C.-G.); guadalupe.mghl@correo.buap.mx (M.G.H.-L.); fermin.flores@correo.buap.mx (F.F.-M.)

**Index**	**page**
Table S1**. Quantitative distribution of cell cycle-treated and signaled.**	2
Image **S1. Quantitative distribution of cell cycle-treated and signaled.**	3
Graphic S1	3
Graphic S2	4
Graphic S3	4
Graphic S4	5
Graphic S5	5
Graphic S6	6
Graphic S7	6
Graphic S8	7
Graphic S9	7
Graphic S10	8
Graphic S11	8
Graphic S12	9
Graphic S13	9
Graphic S14	10
Graphic S15	10
Graphic S16	11

Flow Cytometry Analysis

The analysis of the cell cycle was carried out by flow cytometry after propidium iodide staining. The expression of pTNF-α (sc-133192), pSMAD123 (sc-7960), pMAFbX (sc-166806) and pAKT (sc-514032) was determined by non-phospho-specific antibody in the cells of the four signaling. FSC-A gating was used to collect data, in a BD FACSCanto II.

Statistical Analysis

Signaling Pathway	Treatment	G1 Phase (%)	G2 Phase (%)	S Phase (%)	S Phase Change vs Control
TNF-α	Control	46.59	25.67	27.73	—
	4d	13.87	9.46	76.67	+177%
	Myostatin	35.07	16.04	48.89	+76%
	Myostatin+4d	4.47	9.33	86.20	+211%
AKT	Control	52.46	20.99	26.55	—
	4d	2.18	17.76	80.05	+201%
	Myostatin	4.89	10.42	84.69	+219%
	Myostatin+4d	3.25	14.56	82.19	+210%
MAFBX	Control	20.73	8.33	70.94	—
	4d	3.16	13.39	83.45	+18%
	Myostatin	2.23	9.28	88.48	+25%
	Myostatin+4d	6.36	13.71	79.93	+13%
SMAD123	Control	5.23	18.38	76.39	—
	4d	15.16	7.31	77.53	+1%
	Myostatin	2.15	9.64	88.21	+15%
	Myostatin+4d	14.10	10.33	75.57	-1%

Table S1. Quantitative distribution of cell cycle-treated and signaled.
The % of G1, G2, and S cells in different treatment protocols according to TNF-α, AKT, MAFBX, and SMAD123 pathways were plotted as data. The results represent the average percent of the mean of the representative experiments.

Summary of Key Findings

4d Treatment Effects

Pathway	Baseline S (%)	4d S (%)	Fold Change
TNF-α	27.73	**76.67**	**2.77x**
AKT	26.55	**80.05**	**3.01x**
MAFBX	70.94	**83.45**	**1.18x**
SMAD123	76.39	77.53	1.01x

Synergistic Effects

Pathway	Expected*	Observed	Synergy
TNF-α	62.8%	**86.2%**	**+37%**
AKT	84.7%	**82.2%**	-3%
MAFBX	85.9%	**79.9%**	-7%
SMAD123	81.9%	**75.6%**	-8%

Data interpretation: *Values represent percentage of cells in each cell cycle phase as determined by flow cytometry analysis. S-phase changes are calculated relative to pathway-specific controls. *Expected values for synergy analysis calculated using Bliss independence model (E = EA + EB - EA×EB). Positive synergy indicates supra-additive effects, negative values indicate sub-additive effects.*

Statistical significance: *Data represent mean values from representative experiments. Changes >10% are considered biologically significant based on coefficient of variation in control conditions.*

Color coding: *High effect (>100% increase)* *Moderate effect (20-100% increase)* *Low effect (<20% change)*

Image S1. Quantitative distribution of cell cycle-treated and signaled.

Graphics

Percentages of G1 (in blue), G2 (in red), and S (in green) phases are indicated. Mean of two representative experiments Means ± S.E.

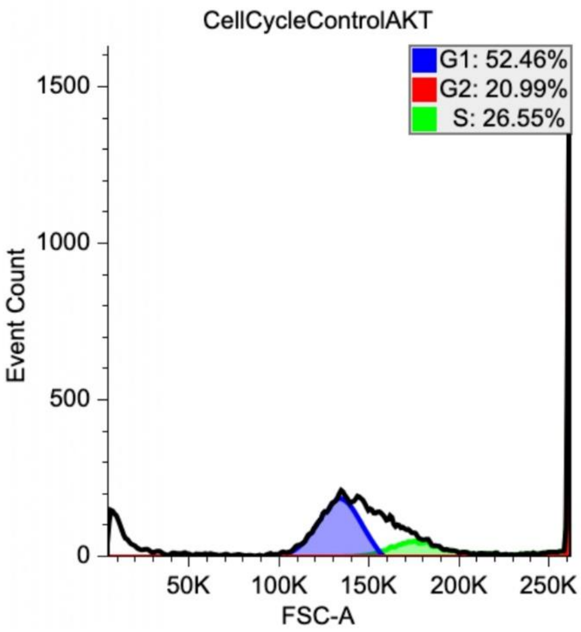

Graphic S1. Control-AKT

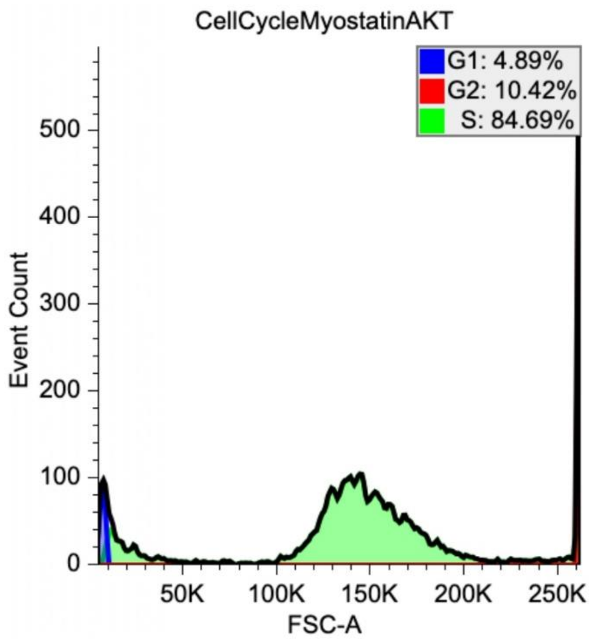

Graphic S2. Myostatin-AKT

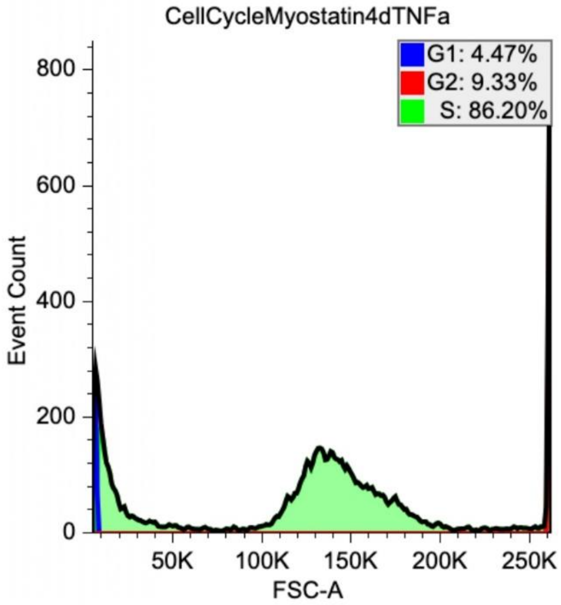

Graphic S3. Myostatin-4d-TNFα

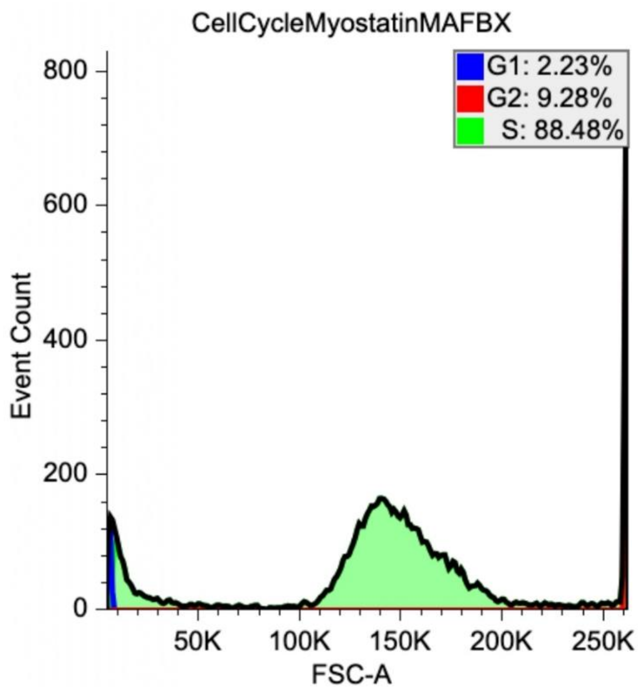

Graphic S4. Myostatin-MAFBX

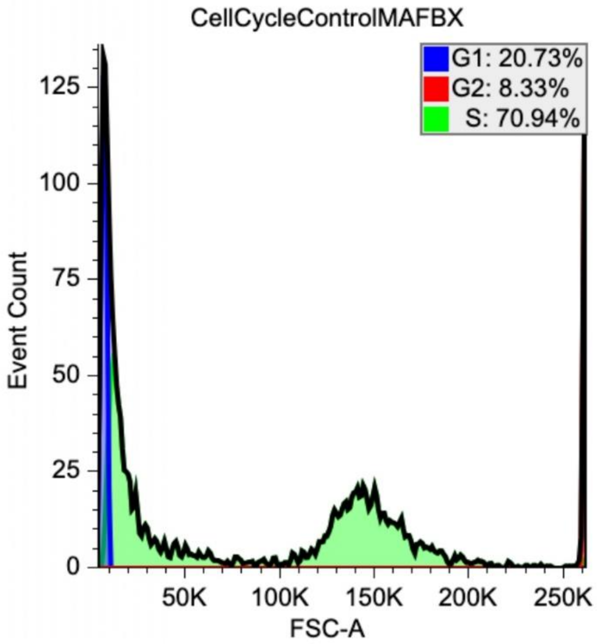

Graphic S5. Control-MAFBX

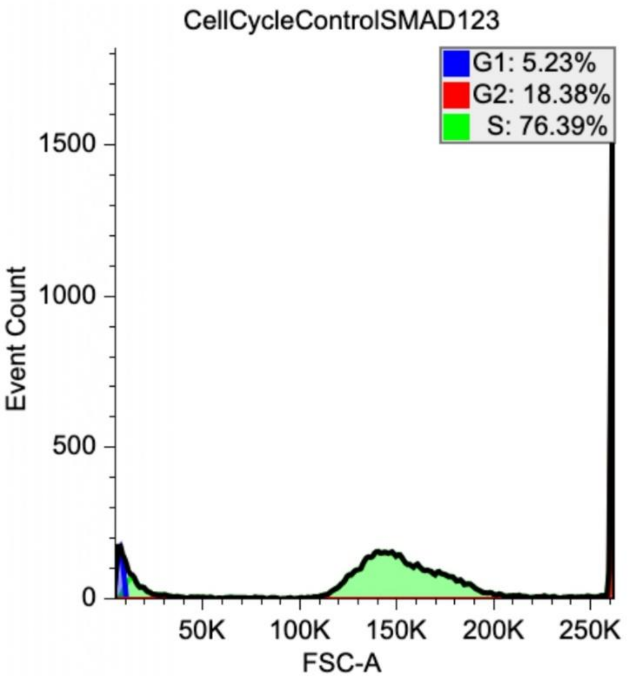

Graphic S6. Control-SMAD123

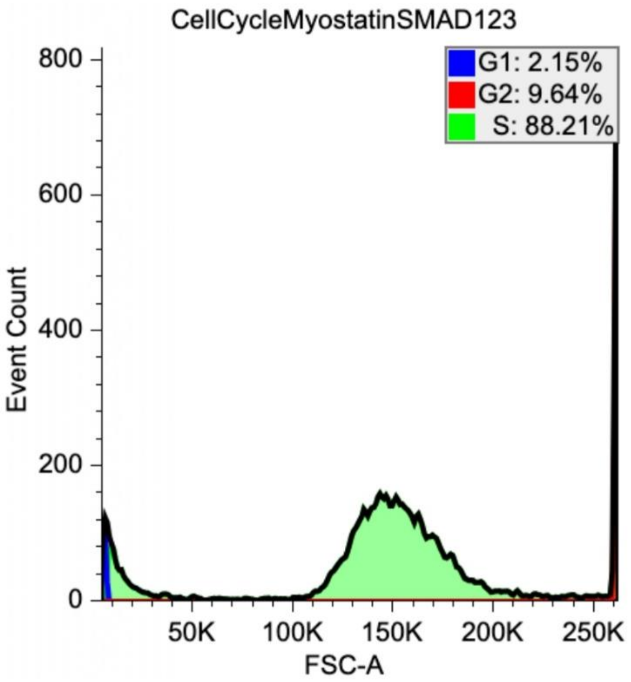

Graphic S7. Myostatin-SMAD123

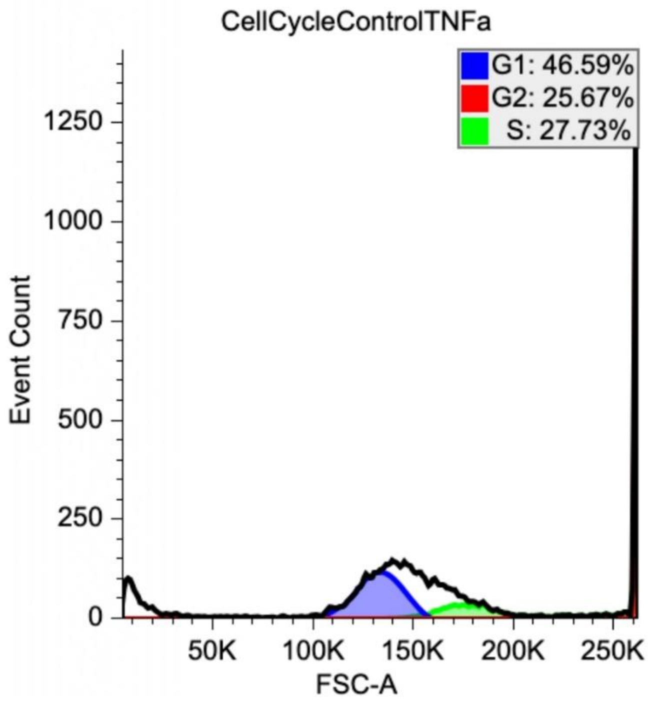

Graphic S8. Control-TNFα

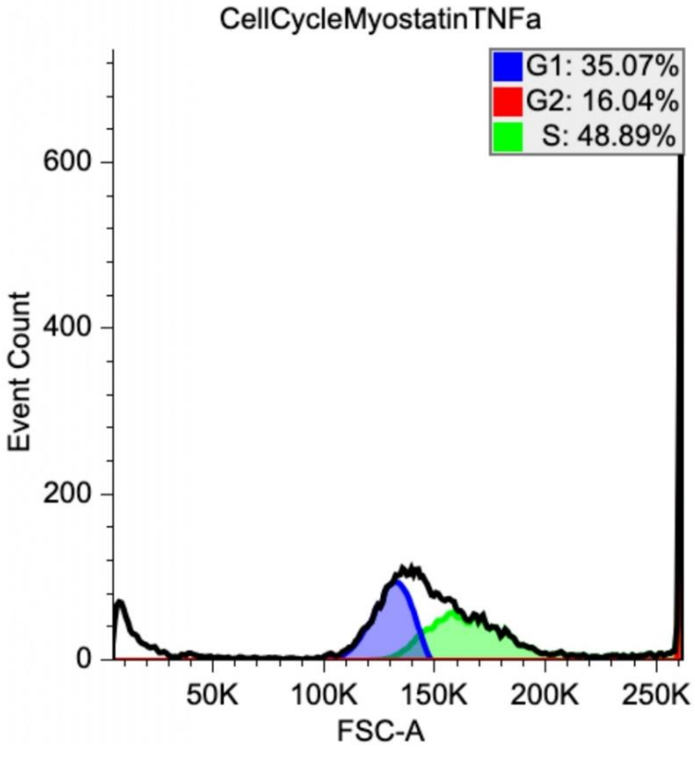

Graphic S9. Myostatin-TNFα

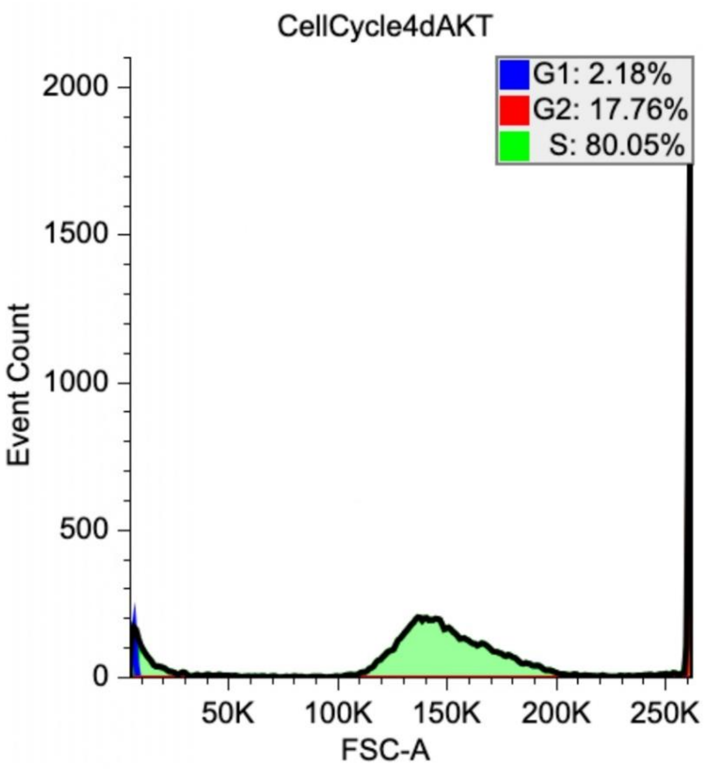

Graphic S10. 4d-AKT

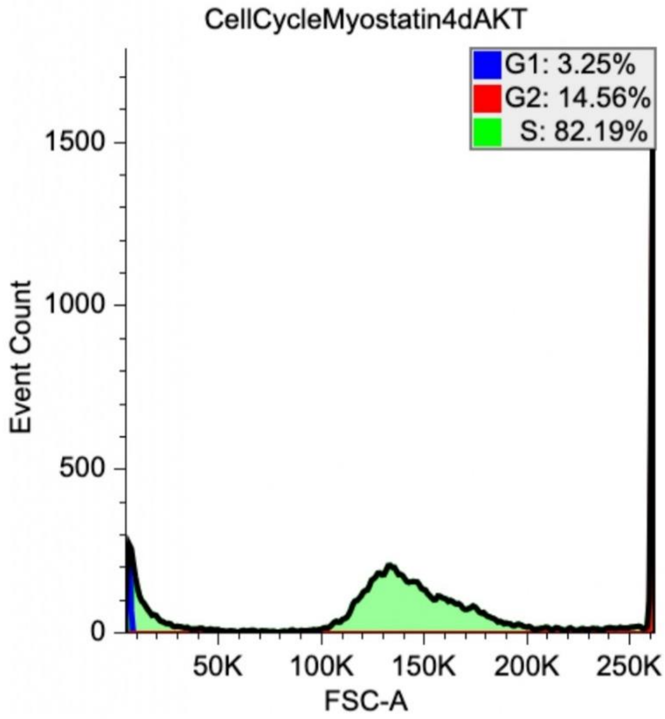

Graphic S11. Myostatin-4d-AKT

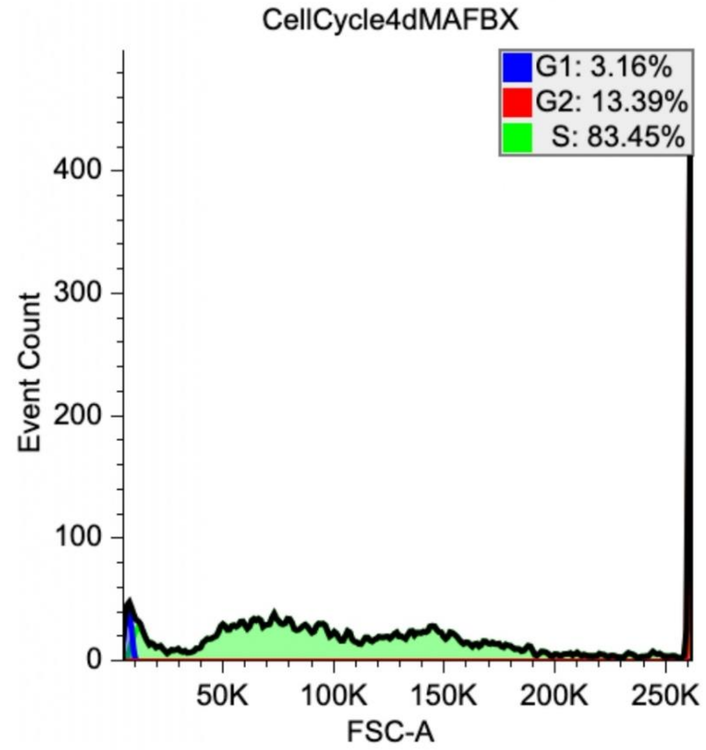

Graphic S12. 4d-MAFBX

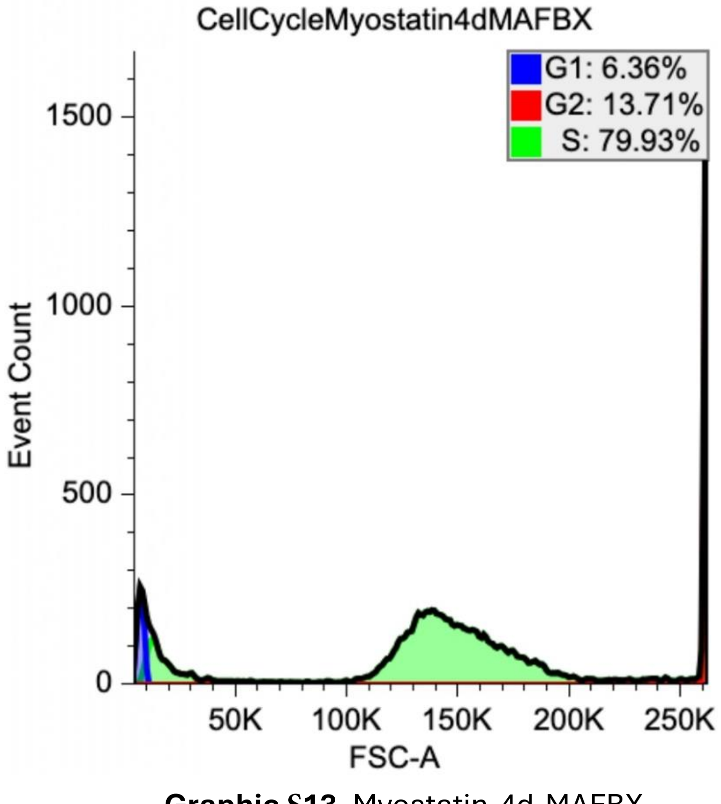

Graphic S13. Myostatin-4d-MAFBX

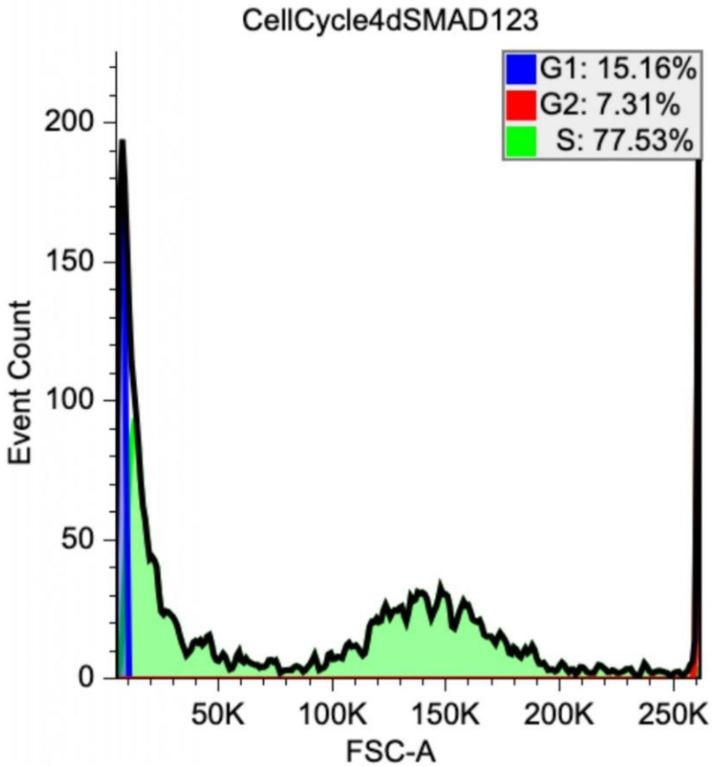

Graphic S14. 4d-SMAD123

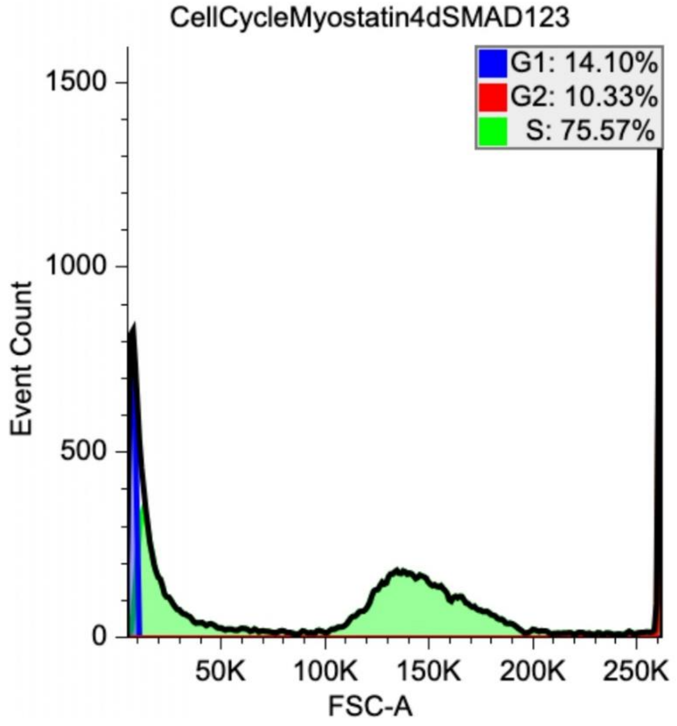

Graphic S15. Myostatin-4d-SMAD123

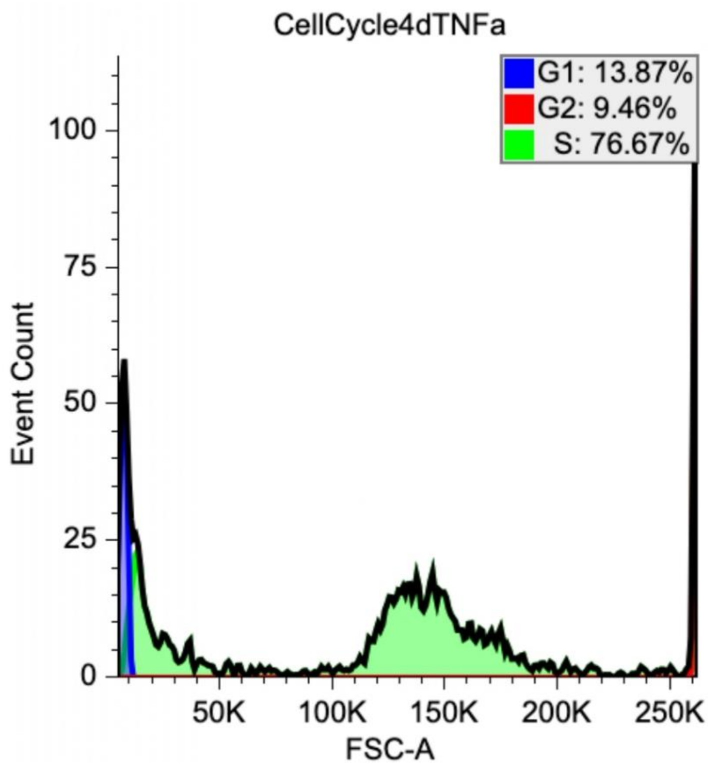

Graphic S16. 4d-TNFα
